# Sex differences in stress and immune responses during confinement in Antarctica

**DOI:** 10.1186/s13293-019-0231-0

**Published:** 2019-04-16

**Authors:** C. Strewe, D. Moser, J.-I. Buchheim, H.-C. Gunga, A. Stahn, B. E. Crucian, B. Fiedel, H. Bauer, P. Gössmann-Lang, D. Thieme, E. Kohlberg, A. Choukèr, M. Feuerecker

**Affiliations:** 1 Department of Anaesthesiology, University Hospital, LMU Munich, Laboratory of Translational Research “Stress and Immunity”, Marchioninistraße 15, 81377 Munich, Germany; 2 Institut für Physiologie, Charité Universitätsmedizin Berlin, Berlin, Germany; 3 NASA - Johnson Space Center, Houston, TX USA; 4 Alfred-Wegener-Institut, Helmholtz-Zentrum für Polar- und Meeresforschung, Bremerhaven, Germany; 5 Institute of Doping Analysis und Sports Biochemistry, Kreischa, Germany

**Keywords:** Sex differences, Neuroendocrine response, Immunity, Extreme environment, Antarctica, Confinement

## Abstract

**Background:**

Antarctica challenges human explorers by its extreme environment. The effects of these unique conditions on the human physiology need to be understood to best mitigate health problems in Antarctic expedition crews. Moreover, Antarctica is an adequate Earth-bound analogue for long-term space missions. To date, its effects on human physiology have been studied mainly in male cohorts though more female expeditioners and applicants in astronaut training programs are selected. Therefore, the identification of sex differences in stress and immune reactions are becoming an even more essential aim to provide a more individualized risk management.

**Methods:**

Ten female and 16 male subjects participated in three 1-year expeditions to the German Antarctic Research Station *Neumayer III*. Blood, saliva, and urine samples were taken 1–2 months prior to departure, subsequently every month during their expedition, and 3–4 months after return from Antarctica. Analyses included cortisol, catecholamine and endocannabinoid measurements; psychological evaluation; differential blood count; and recall antigen- and mitogen-stimulated cytokine profiles.

**Results:**

Cortisol showed significantly higher concentrations in females than males during winter whereas no enhanced psychological stress was detected in both sexes. Catecholamine excretion was higher in males than females but never showed significant increases compared to baseline. Endocannabinoids and *N*-acylethanolamides increased significantly in both sexes and stayed consistently elevated during the confinement. Cytokine profiles after in vitro stimulation revealed no sex differences but resulted in significant time-dependent changes. Hemoglobin and hematocrit were significantly higher in males than females, and hemoglobin increased significantly in both sexes compared to baseline. Platelet counts were significantly higher in females than males. Leukocytes and granulocyte concentrations increased during confinement with a dip for both sexes in winter whereas lymphocytes were significantly elevated in both sexes during the confinement.

**Conclusions:**

The extreme environment of Antarctica seems to trigger some distinct stress and immune responses but—with the exception of cortisol and blood cell counts—without any major relevant sex-specific differences. Stated sex differences were shown to be independent of enhanced psychological stress and seem to be related to the environmental conditions. However, sources and consequences of these sex differences have to be further elucidated.

## Introduction

Antarctica is one of the most remote regions on Earth with a generally misanthropic environment. Expeditioners are exposed to a very cold and harsh climate with a mainly monotonic landscape of snow and ice. This leads, especially during winter, to a complete isolation of sojourners on Antarctic research stations as visiting the sites or an evacuation here are almost impossible. Additionally, the natural day-night rhythm is abrogated as during the summer months the sun never sets which leads to 24 h of daylight and during winter it never rises causing 24 h of almost complete darkness.

It has been demonstrated that this inhospitable environment represents also a suitable and standardized Earth-bound analogue to mimic some factors like monotonous surroundings, repetitive daily routine and confinement, and its possible resulting psycho-social stress that humans are subjected to during long-term spaceflights [1–3]. General findings in respective studies describe hormonal changes due to an altered biological rhythm [4] which directly affects sleep pattern and quality [5–7] as well as psycho-social behavior [8]. Furthermore, endocrine and metabolic dysfunctions and negative mood patterns have been related to these adverse conditions [9, 10], and also altered functions of the innate and adaptive immunity were identified [11–15].

Until recently, most efforts in the field of Antarctic and space research concentrated principally on identifying the impact of such adverse, extreme environment on different aspects of human physiology but did not necessarily focus on identifying potential sex-specific differences or reaction patterns. This was historically due to the male dominance in Antarctic missions. Also in human spaceflight, male dominance has persisted, but in Antarctica, more and more female applicants and crews are recorded. Hence, despite the fact that the quantity of human studies with focus on sex still remains little, research revealed sex-based specificities in different domains of interest in regard to both Antarctic and space exploration missions.

Against the background of stress-related reactivation of latent viruses [16] and an assumed general increased risk for adverse health effects during spaceflight, the sex differences in immune answers already identified in “normal” life might be important to assure crew health. Herein, women present higher CD4^+^ T cell counts, a higher CD4/CD8 ratio, a higher number of B cells, greater antibody responses, and higher immunoglobulin levels [17, 18]. Therefore, women seem to be provided with a greater resistance to and in case of an infection with a more solid and potent immune response but on the other hand more susceptible to autoimmune diseases [19–21]. These findings together with the increasing number of women in Antarctica and in space emphasize the necessity to multiply studies that focus on sex-specific reactions, mechanisms, and outcome and subsequently to install distinctive countermeasures.

Therefore, our study focused on identifying potential sex differences in psychological, neuroendocrine, and immune reactions to the exposition of women and men to the extreme environment of Antarctica as ground-based space analogue at the German *Neumayer III* station during three overwintering campaigns.

On the basis of previous research, we hypothesized that (i) women show a higher susceptibility to psycho-social stress in isolated extreme environments that subsequently triggers an enhanced neuroendocrine response and (ii) thus results in a decreased immune answer in women compared to their male companions.

## Material and methods

### Study environment

The coastal Antarctic research station *Neumayer III* is located in the Atka Bay in the northeast Weddel sea on the Ekström shelf ice at sea level (70° 40′ S/8° 16′ W) and is operated by the German Polar Institute “Alfred Wegener Institute for Polar and Marine Research”, Bremerhaven, Germany. Expeditioners see no sunlight for about 60 days around midwinter (21 June, complete darkness from mid-May to mid-July). Outside temperatures during midwinter phase drop to ~ − 40 °C, while summertime brings temperatures of ~ − 3 °C. These harsh and extreme conditions restrict access to and exit from the station during winter and lead to a complete isolation period of almost 9 months from mid-February till mid-October as also communication via phone or Internet is dependent on current weather conditions. Therefore, all supply goods for the over-wintering period must be provided during summer and stored adequately.

### Study participants

During the expeditions in 2013, 2014, and 2015, in total, 10 women and 16 men took part in the study. Their demographic data are given in Tables 1 and 2. These expeditioners were primarily employed as scientists, cooks, engineers (including IT), electricians, and medical doctors. On-site data acquisition and sample processing were performed by the respective crew surgeon (BF, HB, PG-L). As the medical position in the crew did not necessarily require a scientific background, they all received training in carrying out research protocols and in monitoring the study participants during the experimental period before their deployment.

**Table 1:** Demographic data of the participants of the campaigns 2013, 2014, 2015

	Female	Male
n	10	16
Age [years]	31.8 ± 6.1 (24 - 44)	37.7 ± 9.1 (26 - 55)
Height [m]	1.66 ± 0.06 (1.59 – 1.74)	1.80 ± 0.05 (1.72 – 1.88)^#^
Weight [kg]	67.8 ± 10.0 (59.1 - 88)	86.8 ± 10.7 (71 - 107.7)^#^
BMI [kg/m^2^]	24.5 ± 3.5 (20.5 - 32.3)	26.9 ± 3.4 (22.5 - 33.6)

n = number of participants; BMI = body mass index; data are mean ± SD (range); # significant difference between male and female; p ≤ 0.001 respectively.

**Table 2:** Demographic data of participants separated by sex and winter-over season (WO)

	WO 2013		WO 2014		WO 2015	
Sex	female	male	female	male	female	male
n	4	5	2	6	4	5
Age [years]	30.0 ± 3.7	33.8 ± 2.05	26.5 ± 3.54	36.2 ± 8.95	36.3 ± 6.55	43.4 ± 11.85
Height [m]	1.64 ± 0.07	1.80 ± 0.04	1.69 ± 0.07	1.82 ± 0.05	1.67 ± 0.05	1.76 ± 0.04
Weight [kg]	60.9 ± 1.44	88.6 ± 15.17	64 ± 0	87.2 ± 7.68	76.5 ± 11.24	84.5 ± 10.54
BMI [kg/m^2^]	22.6 ± 1.68	27.4 ± 4.82	22.5 ± 1.88	26.3 ± 2.50	27.4 ± 3.63	27.2 ± 3.22

n = number of participants; BMI = body mass index; data are mean ± SD

### Sample schedule and collection

Baseline data collection (BDC) was conducted in October of the previous year (e.g., October 2012 for the over-wintering campaign 2013) at the Center for Space Medicine and Extreme Environments at the Charité, Berlin, Germany. Generally, all crew members arrived at *Neumayer III* in December of the respective season. First data and sample collection was always performed in February after an “acclimatization” phase and then on a monthly basis till November. Sampling took place in the first week of each month, in the morning around 7:00 am after an overnight fasting period. Physical exercise was not allowed for 24 h prior to sample collection. Psychological data was acquired by paper questionnaires. Saliva samples for cortisol measurements were taken in the morning (7 am) fasted after awakening and in the evening (7 pm) before dinner. Urine samples were collected for 12 h during the day (7 am to 7 pm) and for 12 h during the night (7 pm to 7 am) for catecholamine determination. Blood draw included the sampling of blood into EDTA and lithium-heparinized tubes. After each expedition, all study samples were shipped back to Munich, Germany, at a temperature of at least − 25 °C. Three to 4 months after return from Antarctica, a post data collection (PDC) was conducted at the Center for Space Medicine and Extreme Environments, Charité, Berlin, Germany, or at the Alfred-Wegener-Institute, Bremerhaven, Germany.

### Study outcome parameters and data

The primary outcome parameter to state a potentially enhanced neuroendocrine response was cortisol concentration. Endocannabinoids, catecholamines, the blood cell count, and the scores assessed by psychological tests were defined as secondary outcome measures. Additionally, the cytokine release after various stimulations was defined as secondary outcome parameter to evaluate the immune response.

Parts of the data have been used for a comparison with data from another Antarctic research station in a recent publication [22].

### Study parameters

#### Psychological stress response

Three different paper questionnaires were performed to evaluate and quantify the participants’ emotional stress level and the intensity of perceived anxiety.

##### Current Stress Test (CST)

The test is conceived to assess a person’s acute emotional stress level [23] and mirrors sensitively acute situational changes in subjective stress. It consists of six questions with paired positive and negative adjectives monitoring the perception of current stress (e.g., “tense–calm”). The subjects give their ratings on a 6-point Likert scale. The range for total item means is 1–6, with higher values indicating an increased stress experience. The design of the test reduces the possibility of carry-over effects upon frequent application. The test was applied at BDC, every month during the confinement and at PDC in the morning and evening, respectively.

##### Spielberger State Trait Anxiety Inventory (STAI)

The STAI [24] distinguishes two kinds of anxiety: state anxiety (perceived in a specific situation) and trait anxiety (referring to one’s character). It consists of two parts with each 20 questions rating the answers on a 4-point scale. Global test score ranges between 40 and 160 points. The STAI was evaluated at BDC, every month during the confinement and at PDC.

##### Post-Traumatic Stress Scale-10 (PTSS-10)

The test detects feelings associated with anxiety and depression (e.g., nightmares, sleep disorders, pain). It consists of two parts with part A including yes/no answers to mirror their existence in the last month and part B grading 10 negative feelings in the past few days on a 7-point scale with a total score between 10 and 70 points. The PTSS-10 was applied at three time points (BDC, July, and PDC).

#### Neuroendocrine stress response

##### Cortisol in saliva

Saliva was collected using a Salivette® (Sarstedt, Nümbrecht, Germany). Participants chewed on the cotton swab for 30–45 s which was subsequently frozen and stored at a temperature of at least − 25 °C at *Neumayer III*. Cortisol concentrations were then quantified by an electrochemiluminescence immunoassay (Elecsys 2010, Roche, Mannheim, Germany) at the Institute of Clinical Chemistry, Hospital of the University of Munich, Germany.

##### Urine catecholamines

Norepinephrine and epinephrine were measured from the pooled daytime and nighttime urine by taking a 10-ml sample respectively. The first voided urine volume was discarded before starting the collection. The samples were immediately frozen, stored, and subsequently transported at a minimum temperature of at least − 25 °C. Quantification of catecholamine concentrations was executed at the Institute of Clinical Chemistry, University of Munich, Munich, Germany, using HPLC (Chromsystems, Martinsried, Germany). The absolute mass of excreted catecholamines was determined by multiplication of urine catecholamine concentrations with the respective urine volume.

##### Endocannabinoid (EC) and *N*-acylethanolamide (NAE) measurements from lithium-heparinized blood

EC concentrations were measured from lithium-heparinized whole blood. Samples were drawn from the fastened subject, immediately placed on ice water to prevent temperature effects [25] and after transfer into Eppendorf tubes frozen at a temperature of at least − 25 °C without any delay. Such sample treatment ensures EC stability for at least 6 months [26]. Quantification of the EC concentrations of anandamide (AEA), 2-arachidonoylglycerol (2-AG), and the NAEs oleoylethanolamide (OEA), palmitoylethanolamide (PEA), and stearoylethanolamide (SEA) was executed at the Institute of Doping Analysis und Sports Biochemistry, Kreischa, Germany. The exact method has been described previously [22].

#### Blood analyses and immune cell functions

##### Complete blood cell count from EDTA-anti-coagulated blood

EDTA-anti-coagulated blood samples were used to determine the complete blood cell count using the on-site QBC Autoread plus automated analyzing system (QBC Diagnostics, Port Matilda, PA, USA). Hematocrit, hemoglobin concentration, platelet, and differential white blood cell counts, as well as the percentages of granulocytes and lymphocytes, were quantified.

##### Recall antigen- and mitogen-stimulated cytokine profiles from lithium-heparinized blood

For analysis of secreted T helper type 1/2 (Th1/Th2) cytokine profiles, lithium-heparinized blood was incubated in assay tubes with the same volume of RPMI-1640 (Sigma-Aldrich) and a fungal antigen mixture containing Candida lysate (10 mg/mL; Allergopharma, Reinbeck, Germany) and trichophyton lysate (10 mg/ml; Allergopharma, Reinbeck, Germany) or RPMI-1640 and pokeweed mitogen (PWM) (5 mg/mL; Sigma-Aldrich). PWM acts as a strong “polyclonal” activator, inducing mitosis in lymphocytes in a non-receptor-specific manner. The assay tubes were incubated for 48 h at 37 °C. After incubation, 200 μl of the supernatant were transferred into Eppendorf tubes and frozen immediately at a temperature of at least − 25 °C for future cytokine analyses.

##### Assessment of cytokine production from lithium-heparinized blood

After thawing of the supernatants, concentrations of the cytokines IFN-γ, IL-10, IL-2, and TNF were analyzed by LuminexxMAP® technology (Bioplex®) with commercially available reagents from BioRad Laboratories Inc. (Hercules, CA, USA), according to the manufacturer’s guidelines. Data were analyzed using Bioplex software; the sensitivity threshold was at 2 pg/ml.

### Statistical analysis

The data was tested for normal distribution using the Shapiro-Wilk test. To realize within-group comparisons (e.g. changes in hematocrit over time in one sex), one-way repeated measure analysis of variances (one-way RM-ANOVA) was applied followed by the post hoc Dunnett or Holm-Sidak test to correct for multiple comparisons. Significant differences were determined by comparing baseline time point (BDC) to the deployed and recovery values. Between-group comparisons (between the two sexes) were executed using a *t* test for normally distributed data and the Mann-Whitney *U* test for non-parametric data. The mean differences between time points were considered significantly different if *p* < 0.05 and are indicated as such on each data table and figure. Statistical inferences regarding the interaction of sex and time were based on mixed linear models with a random intercept for each subject, the fixed effects sex, time-point (entered as a categorical variable), interaction of time and sex, age, and BMI and a first-order autoregressive covariance structure. Correlations between parameters were quantified using Spearman’s rank correlation coefficient. Statistical calculations were performed using SigmaPlot® 12.5 (Systat Software, Chicago, IL, USA), IBM SPSS Statistics V.25 (Armonk, NY, USA), and the Statistical Analysis System release 9.4 for Windows (SAS Institute, Cary, NC, USA).

## Results

### Psychological stress response

#### Current Stress Test (CST)

The CST mirrored a low stress level with constant score values below 3 in both sexes in the morning and evening respectively, with no significant differences between the sexes or in the course of the observation period to BDC (Table 3).

**Table 3:** Current Stress Test (CST) and Spielberger State Trait Anxiety Inventory (STAI)

	CST morning		CST evening		STAI state		STAI trait	
	Female	Male	Female	Male	Female	Male	Female	Male
BDC	2.2 ± 0.55	2.4 ± 0.73	2.2 ± 0.79	2.1 ± 0.90	30.0 ± 4.1	32.4 ± 9.9	35.3 ± 6.3	33.8 ± 7.9
February	2.5 ± 1.11	2.7 ± 0.96	2.3 ± 0.99	2.1 ± 0.90	36.4 ± 9.5	36.4 ± 9.6	34.7 ± 7.7	32.6 ± 9.2
March	2.3 ± 0.73	2.3 ± 0.59	2.2 ± 0.94	2.3 ± 0.86	31.2 ± 6.8	32.7 ± 6.4	31.1 ± 6.8	30.9 ± 8.1
April	2.1 ± 0.54	2.7 ± 1.13	2.6 ± 0.76	2.6 ± 1.17	31.7 ± 6.0	36.6 ± 10.8	29.9 ± 4.7	32.3 ± 9.5
May	2.5 ± 1.12	2.2 ± 1.08	2.5 ± 1.28	2.4 ± 1.02	37.6 ± 12.1	33.6 ± 9.7	32.0 ± 7.4	30.9 ± 10.0
June	2.3 ± 0.79	2.4 ± 1.18	2.1 ± 0.71	2.4 ± 1.19	34.4 ± 7.9	33.3 ± 10.6	30.8 ± 7.3	31.7 ± 11.6
July	2.5 ± 1.1	2.4 ± 0.78	2.4 ± 1.0	2.3 ± 0.96	36.3 ± 12.1	34.9 ± 9.3	31.7 ± 6.7	32.3 ± 10.0
August	2.5 ± 1.17	2.3 ± 1.14	2.5 ± 1.23	2.1 ± 0.95	33.4 ± 10.0	34.2 ± 12.0	32.1 ± 8.4	31.6 ± 10.0
September	2.1 ± 0.82	2.3 ± 1.0	2.1 ± 0.9	2.2 ± 1.07	33.4 ± 6.6	34.5 ± 11.8	30.9 ± 5.0	31.8 ± 9.4
October	2.5 ± 1.38	2.4 ± 1.19	2.4 ± 1.02	2.6 ± 1.29	35.5 ± 13.6	35.7 ± 11.7	29.2 ± 5.8	31.8 ± 11.5
November	2.7 ± 1.36	2.7 ± 1.39	2.7 ± 1.55	2.6 ± 1.28	41.0 ± 16.2	38.5 ± 12.2	32.1 ± 10.1	35.5 ± 12.4
PDC	2.3 ± 1.24	2.4 ± 1.06	2.2 ± 1.2	2.4 ± 1.35	35.6 ± 14.0	36.9 ± 12.6	29.4 ± 8.5	32.6 ± 11.6

Data are mean ± SD; units are scores (CST) or points (STAI); female n = 8-10; male n = 13-16; BDC = Baseline Data Collection, PDC = Post Data Collection; no significant changes between females and males or within each group to BDC

#### Spielberger State Trait Anxiety Inventory (STAI)

Scores for both sexes were low throughout the entire observation period and showed no significant differences between the two sexes or within each group. Threshold values indicating anxiety in a specific situation (36.83 ± SD 9.82 [27]) were moderately exceeded by men at the end of the deployment and back in Europe and by females at the onset of the Antarctic winter and in November. Mean total scores displaying anxiety as a character trait (34.45 ± SD 8.83 [27]) were almost never exceeded (Table 3).

#### Post-Traumatic Stress Scale-10 (PTSS-10)

Part A indicated no increased feelings of anxiety in either sex at all time points (data not shown). Part B demonstrated in both sexes a small but not significant increase in negative feelings at each time point (*females n* = 10; BDC 20.9 ± 5.09; July 23.8 ± 10.14; PDC 23.3 ± 12.70; *males n* = 16; BDC 18.88 ± 9.20; July 19.5 ± 7.56; PDC 23.13 ± 10.09).

### Neuroendocrine stress response

#### Cortisol in saliva

**Fig. 1:**
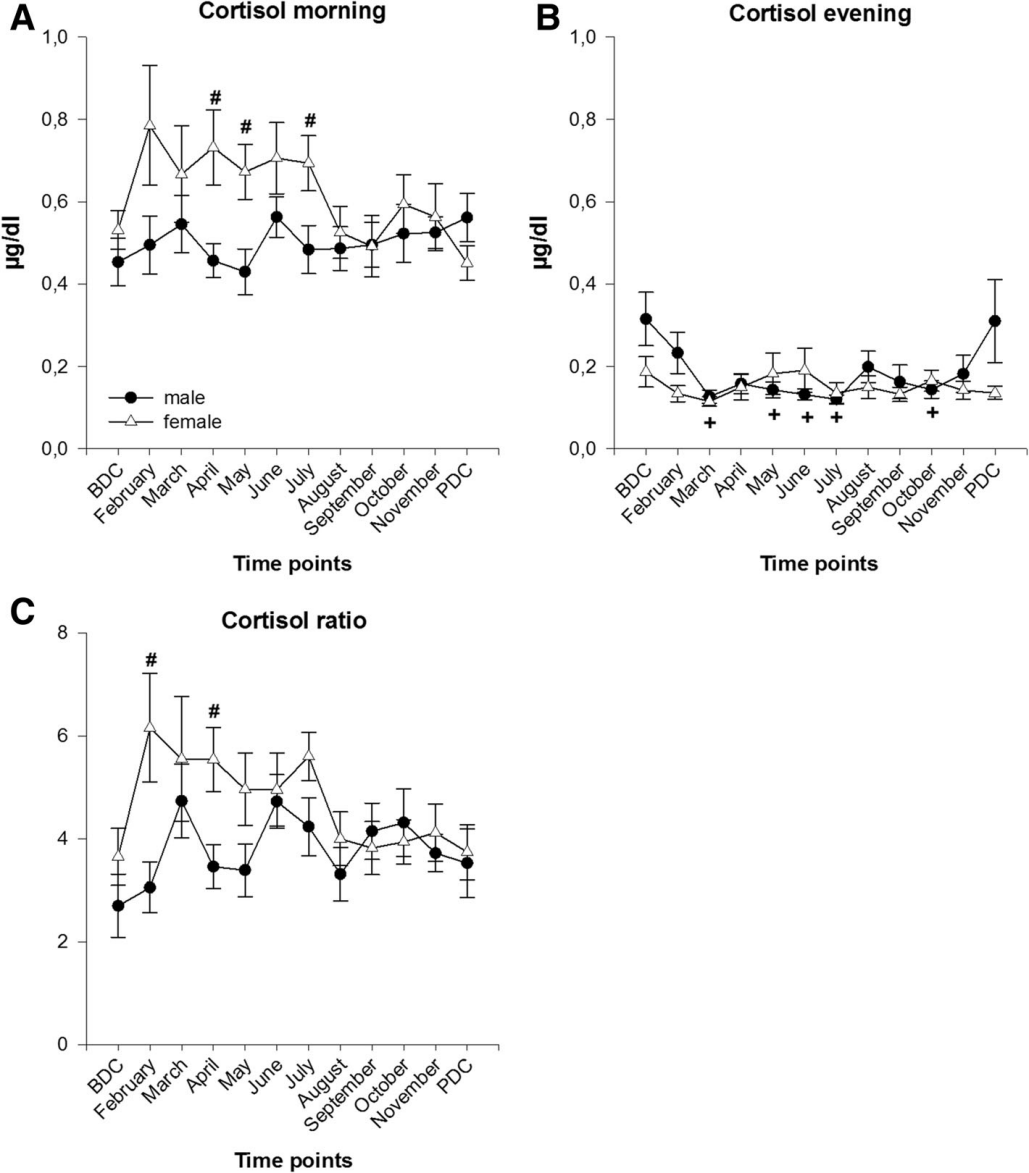
Cortisol in saliva in the morning (**a**) and evening (**b**) and its ratio between morning and evening samples (**c**); data are means ± SEM; units are μg/dl; female *n* = 8–10; male *n* = 13–16. BDC, baseline data collection; PDC, post data collection; #, significant difference between male and female; +, significant difference to BDC in males

Cortisol concentrations in the morning were higher in both sexes at the beginning of the expedition than at BDC even though these changes missed statistical significance. Values in females were consistently higher in April (*p* = 0.009), May (*p* = 0.02) and July (*p* = 0.036) compared to their male colleagues. In the evening, cortisol concentrations dropped in both sexes until March, then fluctuated with no significant differences between males and females. Values in males differed significantly to BDC in March, May to July and October (*F*(11,160) = 2.531, *p* = 0.006) (Fig. 1a, b).

Cortisol ratio between morning and evening values fluctuated but showed constantly higher values throughout the expedition compared to BDC for both sexes. Values in females peaked in February and July and decreased afterwards to baseline values. Values in males peaked in March and June and fluctuated in between. Within-group comparisons showed no statistical significance in males or females, respectively. Between groups comparisons showed a higher cortisol ratio in females than males in February (*p* = 0.02) and April (*p* = 0.017) (Fig. 1c).

#### Urine catecholamines

##### Norepinephrine

**Fig. 2:**
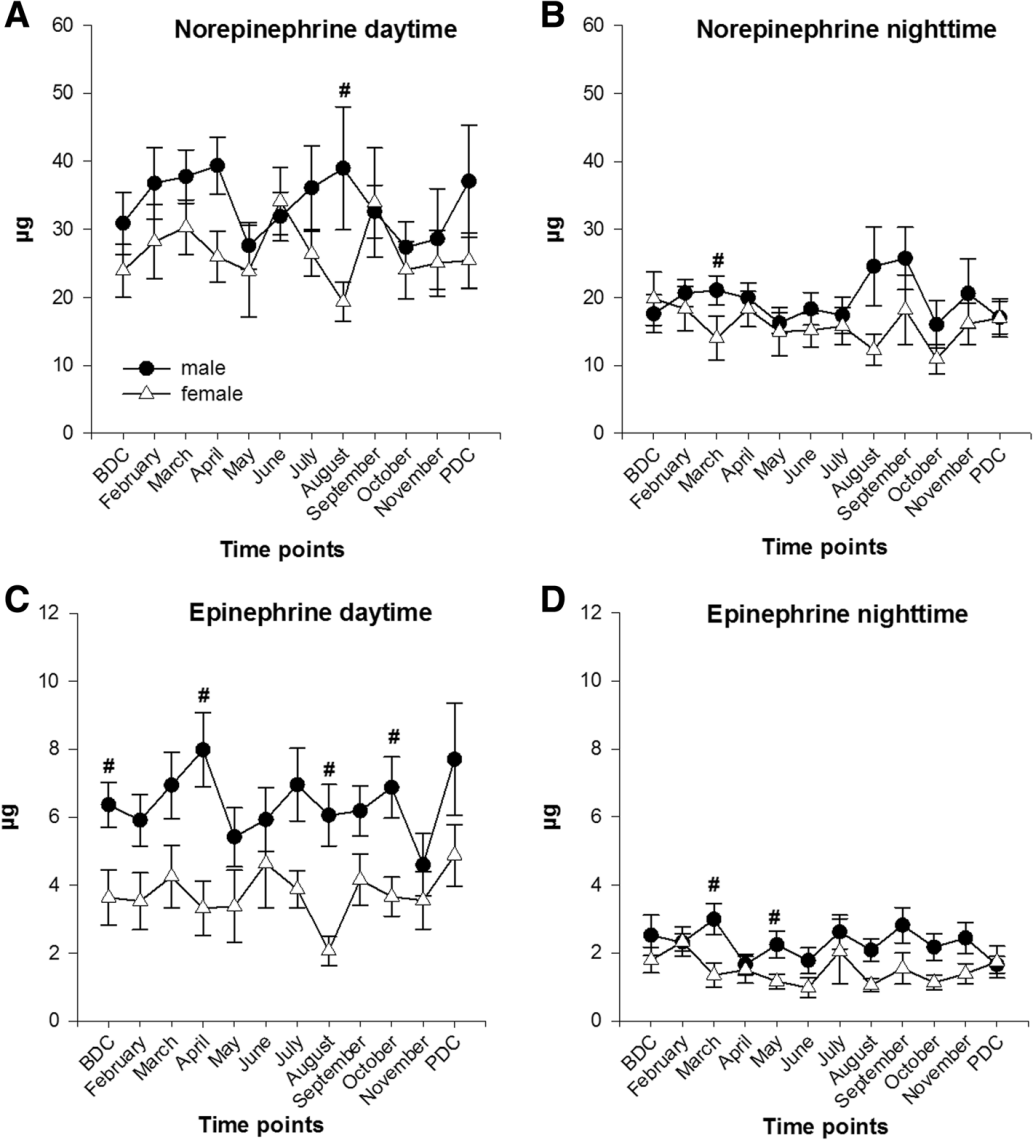
Norepinephrine (**a**, **b**) and epinephrine (**c**, **d**) in urine during the day and night (collection time 12 h); data are means ± SEM; units are μg; female *n* = 6–10; male *n* = 11–16. BDC, baseline data collection; PDC, post data collection; #, significant difference between males and females

In both sexes, norepinephrine excretion during the day showed higher values in the first months of deployment compared to BDC. A drop was found in April/May for both sexes at the onset of the Antarctic winter. In the following months, norepinephrine excretion constantly rose in males to peak again in August whereas female values peaked already in June to subsequently drop to their lowest values in August with a significant difference to their male colleagues’ values (August: *females* 19.35 ± 8.63 μg; *males* 39 ± 36.35 μg; p = 0.01). During the night, norepinephrine excretion was significantly lower in females than males in March (*females* 14.05 ± 10.22 μg; *males* 21.07 ± 8.47 μg; *p* = 0.023). A rise of norepinephrine excretion albeit without any statistical significance was found in both sexes in September (*females* 18.20 ± 16.17 μg; *males* 25.75 ± 18.27 μg) at the end of the Antarctic winter season. In general, the mass of norepinephrine excretion in female expeditioners during the day and the night remained below the values of their male colleagues (Fig. 2a, b).

##### Epinephrine

Total mass epinephrine excretion during the day and the night fluctuated during the deployment period in both sexes. Significant differences between males and females were found at four time points during the day and at two during the night (*epinephrine day*: BDC *p* = 0.02; April *p* = 0.003; August *p* = 0.002; October *p* = 0.014; *epinephrine night:* March *p* = 0.014 and May *p* = 0.047). In general, the mass of epinephrine excretion in female expeditioners during the day and the night remained below the values of their male colleagues (Fig. 2c, d).

##### Endocannabinoids and NAEs

**Fig. 3:**
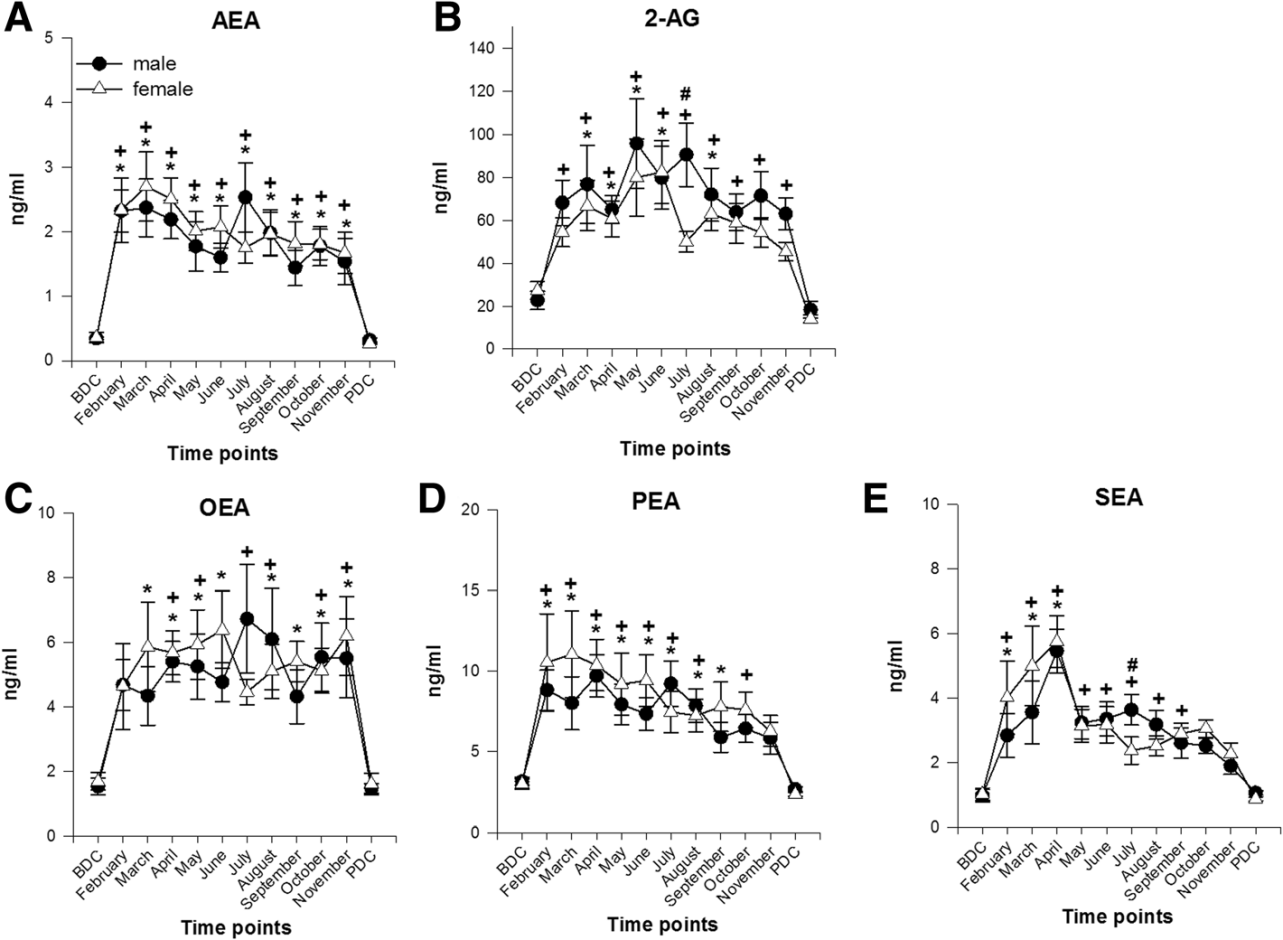
Endocannabinoid and *N*-acylethanolamide concentrations in lithium-heparinized blood. **a**, **b** ECs. **c**–**e** NAEs. Data are means ± SEM; units are ng/ml; female *n* = 9–10; male *n* = 15–16. BDC, baseline data collection; PDC, post data collection; AEA, anandamide; 2-AG, 2-arachidonoylglycerol; OEA, oleoylethanolamide; PEA, palmitoylethanolamide; SEA, stearoylethanolamide; #, significant difference between male and female; +, significant difference to BDC in males; *, significant difference to BDC in females

The ECs AEA and 2-AG and all NAEs increased significantly already in the first months of the Antarctic stay with mean values reaching up to sixfold their basic values (*AEA females F*(11,97) = 6.144, *p* < 0.001; *AEA males F*(11,164) = 5.78, *p* < 0.001; *2-AG females F*(11,97) = 5.224, *p* < 0.001; *2-AG males F*(11,163) = 4.08, *p* < 0.001; *OEA females F*(11,97) = 3.392, *p* < 0.001; *OEA males F*(11,163) = 2.980, *p* < 0.001; *PEA females F*(11,97) = 4.776, *p* < 0.001; *PEA males F*(11,162) = 5.751, *p* < 0.001; *SEA females F*(11,97) = 6.361; *p* < 0.001; *SEA males F*(11,163) = 7.068; *p* < 0.001). This increase was consistent in both sexes. Throughout the observation period, EC and NAE concentrations fluctuated in males and females but always on a highly elevated level and returned to BDC values only when back in Europe. Significant differences between the two sexes were stated for 2-AG and SEA in July (2-AG *p* = 0.016; SEA *p* = 0.048) (Fig. 3a–e).

### Blood analyses and immune cell functions

#### Complete blood cell count (Fig. 4a–f)

##### Hemoglobin

Hemoglobin increased significantly in both sexes during the whole stay in Antarctica and returned to BDC values back in Europe (*females* BDC 13.6 ± 0.6; peak in March 15 ± 1.08 g/dl; *F*(11,93) = 3.008, *p* = 0.002; *males* BDC 15.4 ± 0.73 g/dl; peak in September 16.6 ± 1.43 g/dl; *F*(11,157) = 7.654, *p* < 0.001). Significant differences between males and females were found at all time points with female values being always lower than male values (*p* ≤ 0.001 to 0.005).

**Fig. 4:**
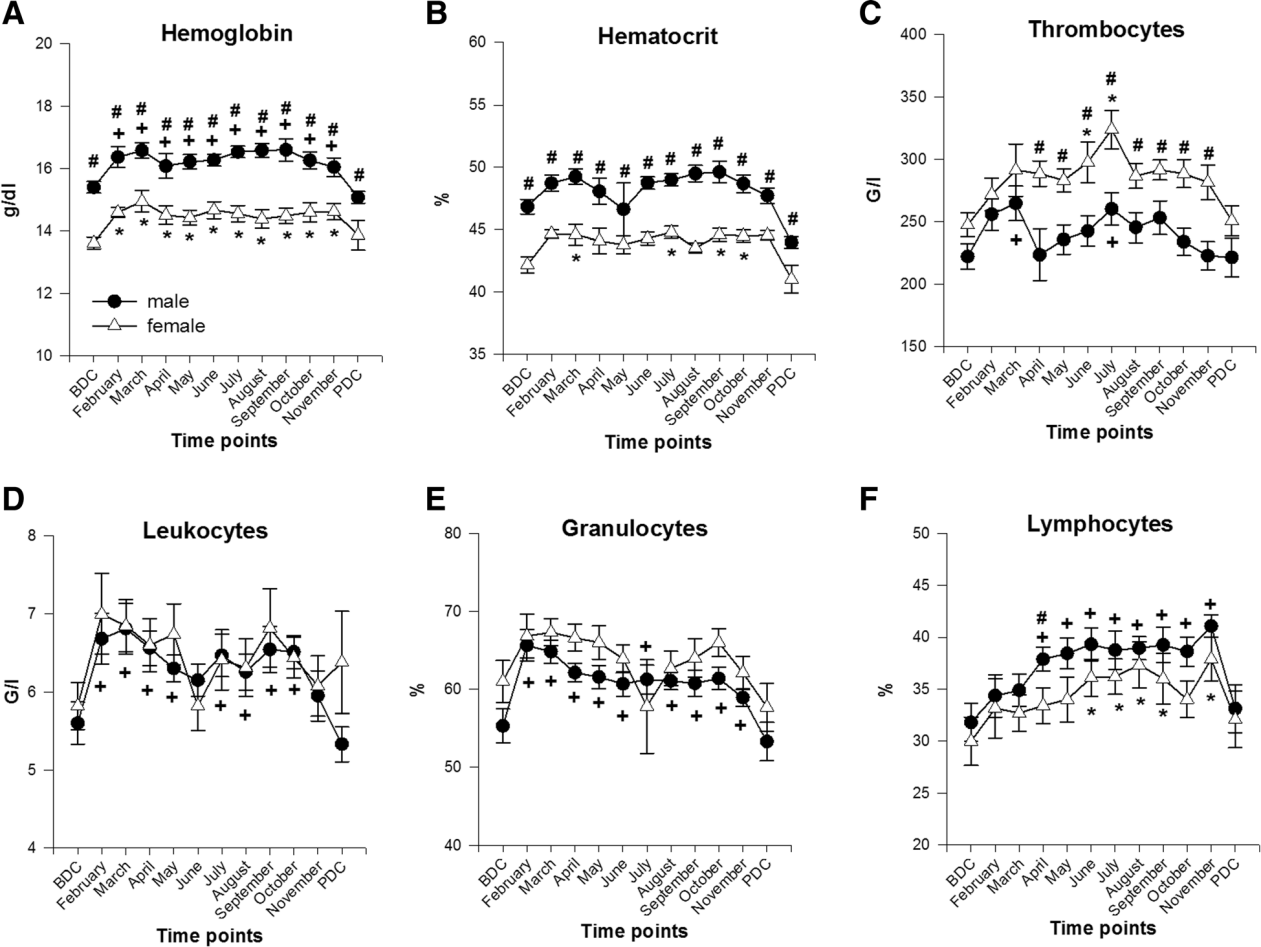
Blood cell count from EDTA anti-coagulated blood. **a** Hemoglobin [units are g/dl]. **b** Hematocrit [units are %]. **c** Thrombocytes [units are G/l]. **d** Leukocytes [units are G/l]. **e** Percentage of granulocytes [units are %]. **f** Percentage of lymphocytes [units are %]; data are means ± SEM; female *n* = 6–10; male *n* = 11–16. BDC, baseline data collection; PDC, post data collection; #, significant difference between male and female; +, significant difference to BDC in males; *, significant difference to BDC in females

##### Hematocrit

Hematocrit showed a similar course to hemoglobin regarding sex differences (significantly different at all time points; *p* ≤ 0.001 to 0.002), but significant changes to BDC were found in females in the months March, July, September, and October (*F*(11,93) = 3.988, *p* < 0.001).

##### Thrombocytes

Thrombocytes increased higher in females with significant differences to males from April to November (*p* = 0.004 to 0.032). Significant differences to BDC were found in females in June and July when their thrombocyte count peaked (*F*(11,93) = 2.961, *p* = 0.002) and for males in March and July (*F*(11,157) = 2.549, *p* = 0.005).

##### Leukocytes

Leukocytes showed a similar course in males and females. They increased in February then fluctuated on an elevated level with male values being significantly different to BDC from February to May and from July to October (*F*(11,158) = 5.862, *p* < 0.001) and returned to baseline values at PDC but stayed elevated in females.

##### Percentage of granulocytes and lymphocytes

The percentage of granulocytes and lymphocytes rose in both sexes but showed almost no statistical in-between differences. In general, percentage of granulocytes was higher in females whereas percentage of lymphocytes was higher in males. Significant differences to BDC for granulocytes were found in males throughout the whole deployment (F(11,158) = 8.455, p < 0.001) and for lymphocytes from April to November (F(11,158) = 6.115, p < 0.001) and for females from June to November (except October) (F(11,93) = 2.238, *p* = 0.018).

#### Recall antigen- and mitogen-stimulated cytokine profiles

##### Basal cytokine release

Basal release of the cytokines INF-γ, IL-10, IL-2, and TNF showed a significant difference to BDC only for IL-2 in female participants in April (*F*(6,53) = 2.287, *p* = 0.049). Female values were consistently lower than male values (Table 4).

**Table 4:** Basal cytokine release for male and female participants

	IFN-γ [pg/ml]		IL-10 [pg/ml]		IL-2 [pg/ml]		TNF [pg/ml]	
	female	male	female	male	female	male	female	male
BDC	0.13 ± 0.10	4.41 ± 10.50	1.14 ± 1.57	7.71 ± 13.63	0.25 ± 0.50	0.15 ± 0.19	0.42 ± 0.48	1.07 ± 1.43
February	0.66 ± 1.78	6.09 ± 17.16	0.88 ± 1.46	11.31 ± 22.80	0.48 ± 1.09	2.87 ± 5.79	0.48 ± 1.23	2.29 ± 6.52
April	0.21 ± 0.35	6.40 ± 15.56	1.97 ± 0.91	#7.89 ± 9.24	*2.76 ± 4.21	3.01 ± 7.33	1.36 ± 1.36	1.84 ± 3.78
June	3.54 ± 7.09	9.80 ± 32.27	6.34 ± 9.57	15.73 ± 26.45	1.21 ± 2.16	2.46 ± 6.37	1.87 ± 3.32	13.20 ± 35.62
August	3.65 ± 7.19	7.54 ± 22.55	2.45 ± 1.34	16.81 ± 28.51	0.76 ± 0.99	2.60 ± 5.65	1.25 ± 1.0	3.73 ± 10.78
October	5.15 ± 8.20	5.68 ± 13.51	2.56 ± 1.55	10.91 ± 18.62	0.98 ± 1.90	3.07 ± 6.28	1.08 ± 0.88	2.30 ± 5.56
PDC	0.10 ± 0	12.73 ± 49.98	2.77 ± 1.34	18.94 ± 34.38	0.33 ± 0.72	1.76 ± 5.19	1.24 ± 0.91	8.80 ± 30.89

Data are mean ± SD; units are pg/ml; IL-2: female n = 9-10; male n = 15-16; IL-10: female n = 5-6; male n = 10-11 ;TNF: female n = 9-10; male n = 15-16; IFN-γ: female n = 9-10; male n = 14-16; BDC = Baseline Data Collection; PDC = Post Data Collection; IL = interleukin; TNF = tumor necrosis factor; IFN-γ = interferon γ; # significant difference between male and female (p = 0.037); * significant difference to BDC in females (p = 0.017)

#### Cytokine profile after stimulation with fungal antigens (Fig. 5a–d)

##### INF-γ

The concentration of IFN-γ rose continuously in both sexes and peaked in June (males) or August (females) but showed significant changes to BDC only for males (June and August). Afterwards, IFN-γ concentrations declined gradually in both sexes to reach baseline levels at PDC.

**Fig. 5:**
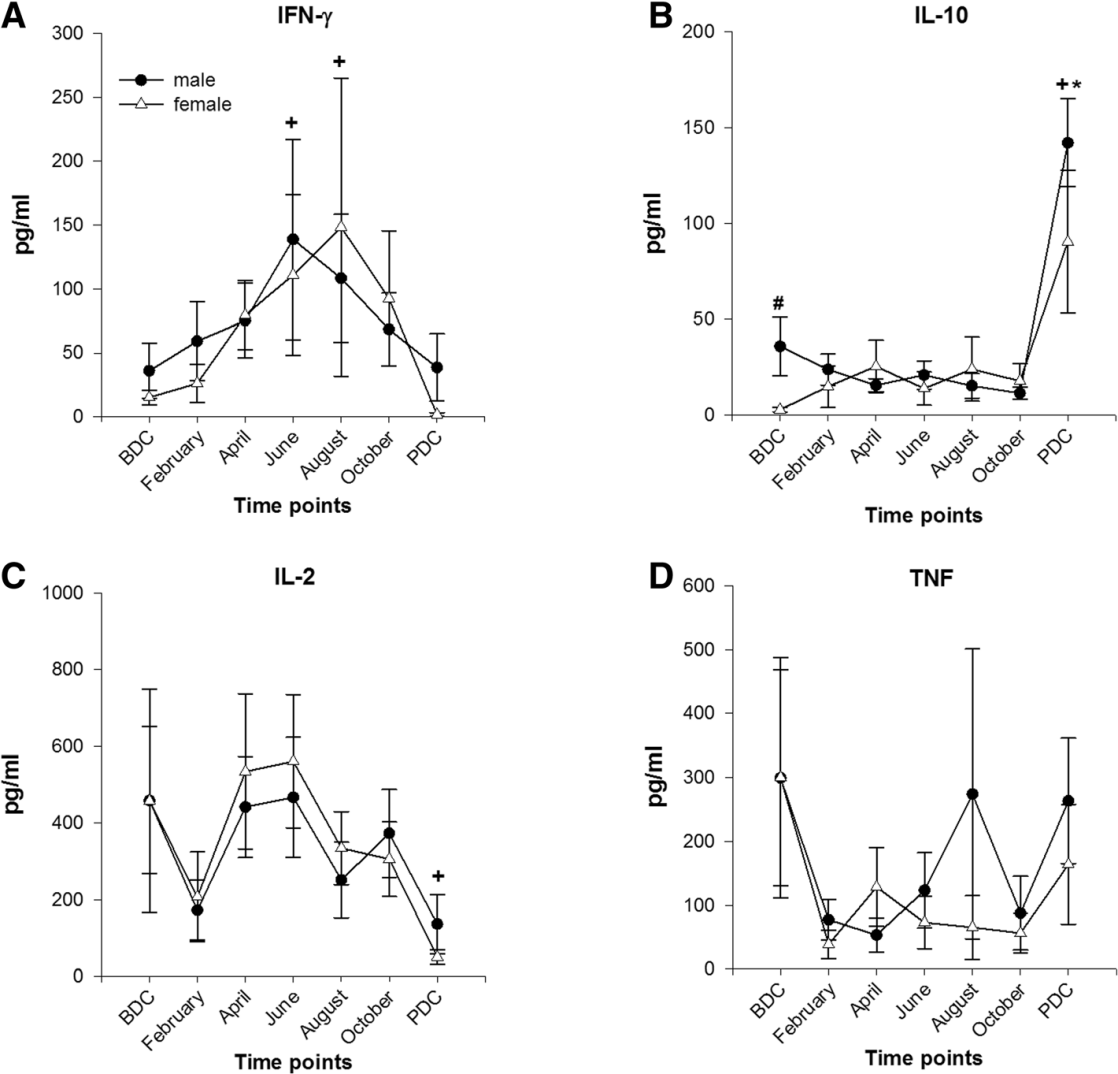
Cytokine release after stimulation with fungal antigens from lithium-heparinized blood. **a** Interferon γ (IFN-γ); female *n* = 9–10; male *n* = 16. **b** Interleukin 10 (IL-10); female *n* = 5–6; male *n* = 11. **c** Interleukin 2 (IL-2); female *n* = 9–10; male *n* = 16. **d** TNF (tumor necrosis factor); female *n* = 9–10; male *n* = 16; data are means ± SEM; units are pg/ml. BDC baseline data collection; PDC, post data collection; #, significant difference between male and female; +, significant difference to BDC in males; *, significant difference to BDC in females

##### IL-10

At BDC, a significant sex difference (*p* = 0.027) was stated that could not be detected in the further course of the observation period. Both sexes showed significant increases in IL-10 concentrations at PBC (females *F*(6,29) = 4.888; *p* = 0.001).

##### IL-2

After stimulation with fungal antigens, the concentration dropped in both sexes in February before it rose to maximum levels in June and declined afterwards with minimum levels at PDC (significant decline to BDC in males). In both sexes, IL-2 levels—although peaking—did only moderately exceed BDC values and never reached statistical significance.

##### TNF

In comparison to BDC, TNF concentration declined during deployment with no difference between sexes. At PDC, TNF levels rose again, however, without reaching BDC values.

#### Cytokine profile after stimulation with the mitogen PWM (Fig. 6a–d)

##### INF-γ

IFN-γ concentrations peaked in April (females) and June (males), but significant differences were only observed in females (April and June, *F*(6,52) = 5.218, *p* < 0.001) compared to BDC.

**Fig. 6:**
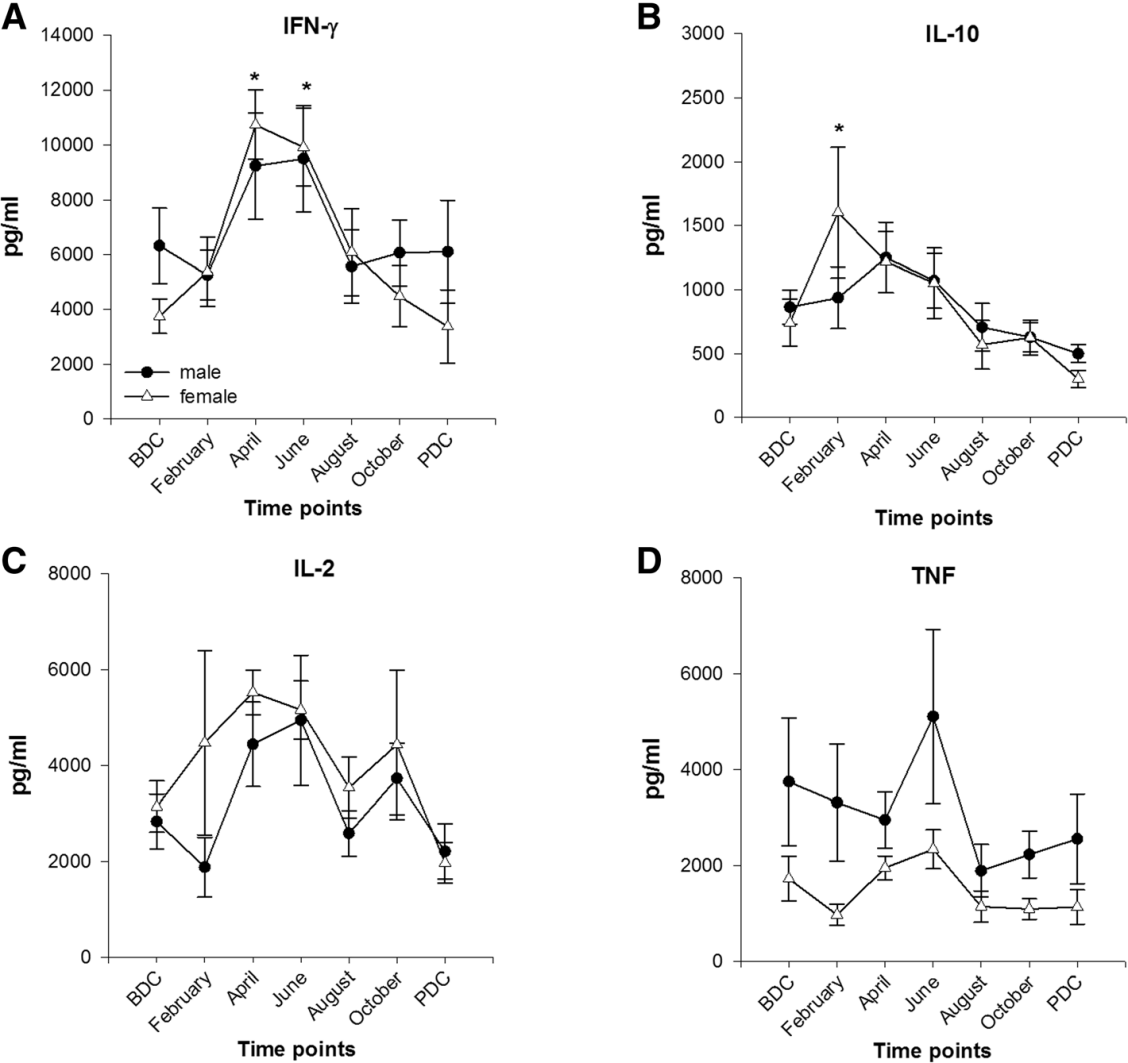
Cytokine release after stimulation with pokeweed mitogen (PWM) from lithium-heparinized blood. **a** Interferon γ (IFN-γ); female *n* = 9–10; male *n* = 16. **b** Interleukin 10 (IL-10); female *n* = 5–6; male *n* = 11. **c** Interleukin 2 (IL-2); female *n* = 9–10; male *n* = 16. **d** TNF (tumor necrosis factor); female *n* = 9–10; male *n* = 16; data are means ± SEM; units are pg/ml. BDC, baseline data collection; PDC post data collection; *, significant difference to BDC in females

##### IL-10

Concentrations increased in females till February and in males till April but only in females the increase was statistically significant (*F*(6,29) = 4.115, *p* = 0.004). Subsequently, concentrations declined continuously with the lowest levels at PDC. No sex differences were observed.

##### IL-2

After an initial drop in February in males, IL-2 concentrations showed continuously higher values with a peak in April (females) and in June (males) compared to BDC, then fluctuated in both sexes and decreased till PDC. No significances were stated.

##### TNF

Till February (females) and April (males), TNF concentrations decreased to subsequently peak in June and then decline below BDC levels till the end of mission albeit without statistical significance.

### General effects of sex, time, and the interaction of sex and time

#### Factor sex

A significant influence of sex was stated for epinephrine (daytime: *p* = 0.0032; nighttime: *p* = 0.008), hemoglobin (*p* < 0.0001), hematocrit (*p* < 0.0001), the percentage of granulo- and lymphocytes (*p* = 0.046; *p* = 0.044), and thrombocytes (*p* = 0.0001).

#### Factor time

All parameters were significantly influenced by the time (*p* ranged between < 0.0001 and 0.04) except epinephrine during the day, cortisol in the morning, IFN-γ after PWM stimulation, the CST (morning and evening), and the STAI (trait).

#### Interaction of sex and time

A significant influence of the interaction of sex and time was found for cortisol in the morning (*p* = 0.012) and for IL-10 after fungal stimulation (*p* = 0.001).

### Correlations between psychological evaluation, neurohormones, and cytokines

#### Correlations between CST and morning cortisol

A positive statistical correlation was found in males (*R* = 0.298; *p* < 0.001) but not in females.

#### Correlations between CST and endocannabinoids

A positive statistical correlation with different endocannabinoids was detected in males (AEA: *R* = 0.183; *p* = 0.022; OEA: *R* = 0.178; *p* = 0.027) and females (OEA: *R* = 0.204; *p* = 0.047; PEA: *R* = 0.216; *p* = 0.035).

#### Correlations between CST and cytokines

A positive statistical correlation with the cytokine IL-10 was found in both sexes (males: IL-10 fungal stimulation *R* = 0.486; *p* < 0.001; females: *R* = 0.604; p = 0.001; males: IL-10 PWM stimulation *R* = 0.36; *p* = 0.007; females: *R* = 0.465; *p* = 0.013).

#### Correlations between morning cortisol and cytokines

A positive statistical correlation with the cytokine IL-10 was detected in males (IL-10 after fungal stimulation *R* = 0.427; *p* = 0.001; after PWM stimulation *R* = 0.428; *p* = 0.001).

## Discussion

The present study focused on the investigation of sex-specific differences in psycho-neuroendocrine and immune responses of humans who were subjected to a 1-year isolation period in the harsh and inhospitable environment of Antarctica at the German Antarctic Research Station *Neumayer III*. Against our hypotheses, sex differences in general were only little. Interestingly, cortisol concentrations in the morning were higher in women than men throughout the winter period however with maintenance of the diurnal rhythm. Opposite to our hypothesis, these elevated cortisol levels in females were not correlated to increased psychological stress and did not specifically result in decreased immune answers in women as based on the assays that could be performed on-site. The few stated positive statistical correlations between psychological evaluation, neurohormones, and inflammatory markers might potentially display their general positive interaction but must be evaluated and interpreted with caution due to the small sample size.

### Differential neurohumoral response pattern and psychological evaluation in males and females

In previous studies, it has been demonstrated that cortisol excretion is influenced by different regulating triggers such as psychological, social, or exercise stress [28–30] and that the cortisol response to specific triggers (e.g., psychological stress) seems to be sex-specific [31]. Furthermore, diurnal cortisol profile, cortisol awaking response and total cortisol excretion over the day are associated with each individual’s chronotype classifying, one as rather a morning or evening type [32–34]. Additionally, recent studies observed that the hypothalamic-pituitary-adrenal (HPA) response to acute psychological and to high-intensity exercise stress is more pronounced in the morning than in the evening, correlating with the circadian rhythm of cortisol synthesis [35, 36]. This finding is in line with our observations of a maintained circadian cortisol rhythm despite higher morning concentrations in women.

Besides psychological and exercise stress, partial or total sleep deprivation, a shorter sleep duration, and also a lower sleep efficiency were observed to be responsible for higher cortisol concentrations [37–39]. Wright et al. [40] assumed the cortisol increase due to sleep deprivation to be a consequence of the absent sleep-induced decrease in cortisol. Moreover, a negative feedback mechanism seems to be serviced by sleep fragmentation inducing increased cortisol levels which, in turn, leads to an increased activation of the HPA axis promoting further sleep fragmentation [41, 42].

Recently, Steinach et al. [43] notably observed a declined sleep quality (increase of time in bed, decline in sleep efficiency, increase in number of arousals) in females during a total of seven overwintering campaigns at the German Antarctic *Station Neumayer II/III* from 2008 to 2014. Against the background of the demonstrated interaction of sleep and HPA activity, these findings support our results of higher cortisol concentrations in females during three overwintering campaigns at *Neumayer III*. Steinach et al. [43] suggested that environmental factors such as isolation, extreme cold, and absence of environmental stimuli might have a higher impact on females than males and discussed a higher susceptibility of women to psycho-social stress to be responsible for the reduction in sleep quality since their physical activity remained unchanged.

However, the psychometric data in our study did not point out any psychological strain of the participants independent of their sex. Furthermore, the hypothesis of an environment triggered cortisol increase and simultaneous decrease in sleep quality is corroborated (i) by the fact of a return of cortisol concentrations in females to baseline levels at the end of the Antarctic winter and (ii) by the results of the examination of the EC system, another stress-related system. It represents a neurobiological mechanism that acts as a regulator in stressful situations and conditions. Activated by physical or emotional stress, it supports the organism in its adaptation to new environmental and physiological challenges [44–46]. In a recently published study of our group [22], we examined the course of EC and NAE concentrations in exclusively male participants of overwintering campaigns at either *Neumayer III* at sea level or *Concordia Station* at high altitude of 3200 m to discern the effects of hypobaric hypoxia. As we detected significantly elevated EC and NAE concentrations during the confinement in the participants at *Neumayer III* but not at *Concordia*, we assumed an activation of the EC system by exposure to the physically challenging environment of Antarctica that is altered and diminished by hypobaric hypoxia at *Concordia.* The actual analysis of the female EC and NAE data at *Neumayer III* revealed the same reaction pattern as stated in their male colleagues which strengthens the assertion of a potential environmental influence. However, in regard to the simultaneously high cortisol concentrations in females, the negative feedback control between the HPA axis and the EC system [47, 48] seems to be blunted in women in this experimental set-up. Sex differences in the HPA axis response to stress have been identified on each level of the regulatory pathway although mostly in animal models [49, 50]. It has been demonstrated that females show a greater HPA axis activation with a greater hormonal response to stress [51, 52], potentially due to a regulatory effect of the gonadal hormones estradiol and testosterone [53, 54]. They have been evidenced to exert both long-term differentiation effects during key developmental periods and also immediate modulatory effects [55, 56]. In humans, research results are more inconsistent and contradicting [57, 58] but the female menstrual cycle seems to play an important role and also the type of stressor or age might have an effect [55, 59, 60].

Additionally, physiological parameters such as the peripheral capillary oxygen saturation (SpO_2_) also seem to succumb these sex-specific influences as Ricart et al. [61] measured at sea level slightly but significantly higher SpO_2_ values in women than in men. Levental et al. [62] confirmed these findings and additionally found no such sex differences in newborns. Thus, the authors assumed that age-related hormonal differences are involved and attributed a possible indirect effect of sex on SpO_2_ to the influence of reduced dead space due to women’s smaller airways. Overall, sex differences seem to appear when specific coexisting influences operate together. In our study, the Antarctic environment apparently creates conditions in which HPA axis activity in women is stimulated.

Moreover, concordant to Steinach et al. [43], physical activity in females stayed balanced during the study period as displayed on the hormonal level by continuously low excreted masses of norepinephrine and epinephrine and therefore has to be excluded as potential explanation for the high cortisol concentrations. The nearly constantly higher masses of both catecholamines in males compared to females, particularly in the daytime urine collection, may be explained by the fact that the physically most strenuous work on-site Antarctic research stations is predominantly done by men (e.g., technicians). A decrease in catecholamine excretion can be stated for both sexes at the onset of the Antarctic winter in April/May and again at the beginning of summer in October/November. This seasonal drop may be explained by a lapse of physical work at the beginning of winter when the crew is more and more restricted to the inside of the station due to weather conditions and at the beginning of summer by the arrival of the summer team that entails a parting of the workload to a larger group of people.

### Sex-related HPA changes and immune effects

To verify our second hypothesis that higher HPA axis activity in women results in a decreased immune response, we analyzed the basal and stimulated cytokine profile after in vitro stimulation with fungal recall antigen and the T and B cell-specific mitogen PWM. A former study of our group [63] demonstrated that this test reflects well the suppressive effects of glucocorticoids on immune responses upon stimulation. However, after analysis, we had to reject our hypothesis as the cytokine profiles between men and women did not vary but rather showed similar courses during confinement. A potential explanation might be that cortisol concentrations were indeed higher after arrival in Antarctica in females compared to BDC but nevertheless never exceeded normal ranges (reference values according to the manufacturer: morning < 0.87 μg/dl; evening < 0.35 μg/dl).

In the stimulation assay, basal values of all measured cytokines were close to detection limit and demonstrated no systemic inflammation for either sex (stated statistical significances are probably due to single identified outliers). Furthermore, no episodes of clinical infection or acute illness were stated or reported during each of the three campaigns. Remarkably, stimulation with fungal antigens and PWM resulted in a significant increase of the pro-inflammatory cytokine IFN-γ in both sexes during the Antarctic winter and a subsequent decrease and return to baseline values till PDC. Simultaneously, the anti-inflammatory cytokine IL-10 showed no peculiar reaction pattern upon stimulation during the confinement but fungal recall antigen exposition resulted in a significant increase in both sexes after the return to Europe at PDC. A former study presented similar results with elevated plasma IFN-γ and suppressed IL-10 concentrations however without a specific time point after return to “normal life” conditions [64]. Furthermore, lately, we [12] published data showing increases in IFN-γ, IL-2, and TNF concentrations after the same recall antigen stimulation assay with peak concentrations after 4 to 7 months of confinement in expeditioners at the Antarctic Research Station *Concordia*. In difference to the present study, cytokine release remained elevated during the whole expedition and did not decrease after winter which might be explained by the immune modulating effect of hypobaric hypoxia that reigns at *Concordia Station* due to its high altitude location.

Therefore, in addition to the EC measurements, also our observations concerning the reactivity of the immune system corroborate the assumption that the Antarctic environment and in particular the winter period seem to affect the human immune system. However, in contrast to the HPA axis reaction, sex differences do not seem to play a significant role here. During winter when expeditioners are confined to the station and immunological challenge and sensitization is low, the immune system seems to stand at attention which might lead to its boost reactivity after re-exposure to a “normal” microbial load.

Restrictively, it has to be stated that the immune answers were not totally consistent to that effect that other pro-inflammatory cytokines such as IL-2 and TNF showed less distinct profiles than IFN-γ. TNF showed a reduction upon fungal stimulation during confinement. However, due to the high inter-individual variability especially at BDC, the data has to be evaluated with caution. One might only speculate that monocytes and macrophages—the main TNF-producing cell lines [65, 66]—are restricted in their functioning after fungal stimulation in this setting. This could also explain a less effective answer after stimulation with the T and B cell-specific mitogen PWM. Additionally, blood cell counts showed an increase in leukocytes and granulocytes in both sexes (statistically significant only for males) with an m-shaped curve during the expedition with a dip during midwinter. Simultaneously, lymphocytes increased significantly in both sexes over time. Similar results were presented by Yi et al. [67], and they speculated that in the absence of immune challenges during isolation, the immune system might release a disproportionately high number of lymphocytes in the peripheral system to prepare for a potential infection. Moreover, different stressors (e.g., low temperature, isolation) seem to activate the physiological stress response to a different extent [68]. Although an animal model, Bowers et al. [68] stated stressor-specific alterations in corticosterone and immune responses with low temperature leading to a significant increase of corticosterone and trafficking of lymphocytes and monocytes but without an enhanced delayed-type hypersensitivity (DTH) response. Additionally, recent human studies [69, 70] demonstrated that sex-specific cold responses induce different neuroendocrine, immune, and memory responses in men and women. However, the effect of these alterations (immune suppression vs. activation) is not distinct and varies depending on the respective study [71–73].

In summary, our observations give further hints to support the assumption of a generally alerted immune system with a shift towards a lymphocyte-mediated answer even though the absolute count of the different cell lines remained in normal ranges.

### Sex differences in blood cell count

Expected sex differences were found for hemoglobin and hematocrit with higher values in males than females as of common knowledge [74, 75]. However, a definite plausible explanation for the significant increase in hemoglobin in both sexes during the isolation period remains missing. Low air pressure and subsequently reduced oxygen partial pressure can be excluded as potential reason, as *Neumayer III* is situated at sea level with an air pressure of ~ 980 mbar. One potential cause could be fluid shifts as hematocrit also increases albeit not constantly significant which might indicate a relative volume loss.

Interestingly, platelet count in women increased significantly in winter compared to BDC and values were significantly higher than in males nearly throughout the whole observation period. Former studies already demonstrated higher platelet counts in women [76, 77] but showed discordant results with higher or lower platelet reactivity in women dependent on the existent general disease [76–78]. Furthermore, there seems to be a relation between endogenous corticoid concentrations and the risk of venous thromboembolism [79, 80]. Here, one might speculate that thrombocyte count plays perhaps a role. Against this background, the sex differences in platelet count might be associated with the higher cortisol concentrations in females but might also be due to a sex-specific reactive sensitivity under exposure to the environmental challenges in our study set-up.

For verification and more substantiated statements, further and more detailed and precise examination of the different cell lines and immune components with focus on sex differences are necessary. The same accounts for the identification of their triggers.

Due to blood volume restrictions and overall difficult research conditions in this field study, a closer look in cell differentiation and more specific immune cell functional states was unfortunately not possible.

### Conclusion

In summary, we conclude that Antarctica with its extreme environment as an Earth-bound analogue for long-term space exploration class missions seems to trigger some distinct physiological answers but without eliciting major relevant sex-specific differences in these answers. Detected sex differences were related to a higher HPA axis activity and a higher platelet count in females than males, even though their consequences remain unknown and need to be identified in future studies. Moreover, we had to reject our hypotheses of higher cortisol concentrations in females (i) being caused by higher psychological stress and (ii) resulting in a decreased immune answer (as detectable with the performed tests) as women showed the same psychological resilience and immune responses as males. Instead, the examination of other stress-related systems such as the EC system provided further evidence to support the explanation of the environment being the trigger for our sex-specific and general observations.

On the long run, more detailed and precise sex- and trigger-oriented studies will support a better individual risk assessment and the development of appropriate countermeasures for both male and female Antarctic expeditioners and space travelers.

## Limitations

Sample collection, processing, and functional tests were performed by the expedition’s medical doctors who were no specialists in laboratory research and had to perform under extreme experimental conditions. A more cell-differentiated immune analysis and, further, more detailed functional tests with regard to potential sex differences were not possible due to tight blood volume and financial restrictions that entailed the absence of a flow cytometer on-site. Due to overall limited cases, a one-to-one correlation was also not possible.

To validate the assumed environmental effects on human physiological answers and to verify the stated sex differences a control group exposed to normal life conditions or even a cross-over study design would have been necessary and ideal. However, such study designs are barely feasible in extreme environment field studies with regard to particularly logistics, costs, participants, and others. Additionally, it must be stated that the participants of such long-term, difficult, and also dangerous expeditions pass through a rigorous selection process and experience a specific training before departure which aims to prepare them for potential conflict situations not only physically but also psychologically. Therefore, the study group misses to be an exact representative of the general population which might have an influence on measured outcome parameters. Furthermore, a more specific and defined study set-up concerning sex differences would improve the outcome of isolating and distinguishing them from general alterations.
